# Correlation between tooth size-arch length discrepancy and interradicular distances measured on CBCT and panoramic radiograph: an evaluation for miniscrew insertion

**DOI:** 10.1590/2177-6709.23.5.39.e1-13.onl

**Published:** 2018

**Authors:** Michele Tepedino, Marie A. Cornelis, Claudio Chimenti, Paolo M. Cattaneo

**Affiliations:** 1 University of L’Aquila, Department of Biotechnological and Applied Clinical Sciences (L’Aquila, Italy).; 2 Aarhus University, Faculty of Health, Department of Dentistry and Oral Health, Section of Orthodontics (Aarhus, Denmark).

**Keywords:** Orthodontics, Orthodontic anchorage procedures, Orthodontic miniscrew, CBCT, Safe zones

## Abstract

**Introduction:**

The selection of appropriate sites for miniscrew insertion is critical for clinical success.

**Objectives:**

The aim of the present study was to evaluate how interradicular spaces measured on panoramic radiograph compare with Cone-Beam Computed Tomography (CBCT), and how crowding can influence the presence of available space for miniscrew insertion, in order to define a new “safe zones” map.

**Methods:**

A total of 80 pre-treatment panoramic radiographs and 80 CBCT scans with corresponding digital models were selected from the archives of the department of Dentistry, Aarhus University. Crowding was measured on digital models, while interradicular spaces mesial to the second molars were measured on panoramic radiographs and CBCTs. For panoramic radiographs, a magnification factor was calculated using tooth widths measured on digital models. Statistical analyses were performed to investigate the correlation between the amount of crowding and the available interradicular space. Visual maps showing the amount of interradicular spaces measured were drawn.

**Results:**

The most convenient interradicular spaces are those between the second molar and the first premolar in the mandible, and between the central incisors in the maxilla. However, some spaces were revealed to be influenced by crowding.

**Conclusions:**

Calibration of panoramic radiographs is of utmost importance. Generally, panoramic radiographs underestimate the available space. Preliminary assessment of miniscrew insertion feasibility and the related selection of required radiographs can be facilitated using the new “safe zone” maps presented in this article.

## Resumo

**Introdução:**

a seleção de locais apropriados para a inserção de mini-implantes é crítica para o sucesso clínico.

**Objetivo:**

os objetivos do presente estudo foram avaliar as medições de espaços inter-radiculares feitas em radiografias panorâmicas e compará-las com as medições feitas com tomografia computadorizada de feixe cônico (TCFC), além de avaliar como o apinhamento pode influenciar na disponibilidade de espaços para inserção dos mini-implantes, no intuito de definir um novo mapa de “áreas seguras”.

**Métodos:**

foram selecionadas, nos arquivos do departamento de Ortodontia da *Aarhus University*, 80 radiografias panorâmicas pré-tratamento e 80 imagens de TCFC com os modelos digitais correspondentes. O apinhamento foi medido nos modelos digitais, enquanto os espaços inter-radiculares mesiais aos segundos molares foram medidos nas radiografias panorâmicas e na TCFC. O fator de magnificação das radiografias panorâmicas foi calculado utilizando-se as larguras dentárias medidas nos modelos digitais. Análises estatísticas foram realizadas para investigar a correlação entre a quantidade de apinhamento e o espaço inter-radicular disponível. Foram desenhados mapas visuais mostrando a quantidade dos espaços inter-radiculares medidos.

**Resultados:**

os espaços inter-radiculares mais adequados são aqueles entre o segundo molar e o primeiro pré-molar inferior, e entre os incisivos centrais superiores. Porém, verificou-se que alguns espaços são influenciados pelo apinhamento.

**Conclusões:**

A calibração das radiografias panorâmicas é de suma importância, pois, geralmente, as radiografias panorâmicas subestimam o espaço disponível. A avaliação preliminar da viabilidade de inserção dos mini-implantes e a seleção das radiografias necessárias para isso podem ser facilitadas utilizando-se os novos mapas de “áreas seguras” aqui apresentados.

## INTRODUCTION

Orthodontic miniscrews are devices specifically designed to be temporarily inserted into the alveolar bone to enhance anchorage.^1^ They are commonly used when patient compliance is an issue, when the number of teeth does not allow appropriate anchorage, or when teeth are periodontally compromised.^2^ The success rate of orthodontic miniscrew insertion is reported to be between 61% and 100%,^3^ ^,^ ^4^ being affected by many factors;^1^ ^,^ ^3^ ^-^ ^7^ and root proximity appears to play an important role.^8^ The choice of appropriate insertion site is critical: it is important to place the miniscrew in a site that is convenient from a biomechanical point of view, and to do so without damaging any surrounding periodontal structures (dental roots, maxillary sinus, nerves) during the insertion procedure. Many authors have defined maps of “safe zones” for miniscrew insertion; a few studies were conducted on panoramic radiographs,^9^ ^,^ ^10^ whilst the majority used Cone Beam Computed Tomography (CBCT) to determine the “quality” and suitability of different insertion sites.^11^ ^-^ ^20^ The former are affected by horizontal and vertical magnifications^21^ ^-^ ^24^ typically caused by the patient’s positioning, tooth angulations and root positions, and degrees of asymmetries within and between the jaws.^9^ ^,^ ^21^ ^,^ ^25^ Still, they represent a simple, low-cost and low-dose radiographic examination routinely prescribed to orthodontic patients.^26^ CBCTs, on the other hand, represent the gold standard in 3D radiographic imaging due to a relatively low radiation dose and high-quality images, which provide more realistic images than 2D images.^27^ However, according to previous studies, there is little consensus regarding how much information CBCTs can provide over conventional radiographs, and in which cases increased radiation exposure can be justified.^27^ ^-^ ^32^

Schnelle et al.^9^ investigated the presence of 3 and 4 mm of space between two adjacent roots on panoramic radiographs in order to define a map of interradicular sites where a miniscrew could be safely placed. This amount of space was chosen as a typical miniscrew diameter is between 1.2 and 2 mm, and there must be at least 1 mm of bone surrounding the miniscrew to avoid root damage during insertion. Other authors measured the space between the roots at different heights on panoramic radiographs^10^ or CBCTs.^13^ ^,^ ^14^ ^,^ ^16^ ^,^ ^18^ ^,^ ^20^ ^,^ ^33^ A systematic review of the available literature concluded that ideal sites for orthodontic miniscrew placement, defined by appropriate quantity and quality of bone, are the buccal and lingual interradicular spaces between the second premolar and the second molar, both in the maxilla and the mandible.^11^

Another possible parameter for identifying miniscrew insertion sites is dental crowding. Different malocclusions show differences in bone availability between the roots due to dentoalveolar compensation of skeletal discrepancies.^12^ Moreover, Schnelle et al^9^ found increased interradicular space for miniscrew insertion after tooth alignment, compared with before treatment. However, it has not previously been demonstrated whether there is any correlation between the amount of interradicular space and dental crowding. 

Landin et al^35^ compared the percentage of miniscrews placed without damaging surrounding structures by blind insertion with having either a pre-operative periapical radiograph, a panoramic radiograph or a small-volume CBCT. Interestingly, blind placement, periapical radiograph and panoramic radiograph performed almost the same, suggesting that pre-operative 2D radiographic images give no significant advantage. On the other hand, three-dimensional information provided by CBCT was significantly more useful, though at the cost of an increased ionizing radiation dose.

A miniscrew insertion site evaluation method that would minimize or even discard the use of ionizing radiation would be very advantageous for both clinicians and patients in light of the ALARA (As Low As Reasonably Achievable) principle. For this reason, the present paper addresses the following question: Is there a way of increasing the amount of information extrapolated from a traditional radiograph by combining it with specific clinical observations to ensure safe miniscrew placement at low radiation cost? The goal is to help the clinician from the first step of his orthodontic treatment planning, choosing the appropriate clinical and/or radiological examination, in order to determine the possible insertion site(s) of miniscrews.

Therefore, the aims of this study were to evaluate: 1) how the assessment of interradicular spaces on panoramic radiographs performs compared with CBCTs; and 2) how the presence of radiologically adequate interradicular spaces correlates with tooth size-arch length discrepancy, in order to define a new “safe zones” map. The hypothesis was that the amount of tooth size-arch length discrepancy can be used as a pre-treatment clinical tool to assess the possibility of miniscrew insertion.

## MATERIAL AND METHODS

Permission to use the material for this retrospective study was granted by the Danish Data Protection Agency (Aarhus University Journal no. 62908). Eighty pre-treatment panoramic radiographs (Panoramic group) and eighty CBCT scans (CBCT group) of patients previously treated at the Section of Orthodontics, Aarhus University were randomly selected (www.randomizer.org) from the archive according to the following inclusion criteria:

» Permanent dentition, with second molars erupted.
» No agenesis or missing teeth, except for third molars.
» Patients under 35 years of age at the time of the radiographic examination.
» Absence of signs of periodontitis and/or bone resorption on the radiographic examination.
» Pre-treatment digital models available.

The radiographic records were retrieved from the archive of previously treated patients; they had been taken in accordance with the radiological guidelines of the Department of Dentistry, Aarhus University.

### Digital models

**Figure 1:**
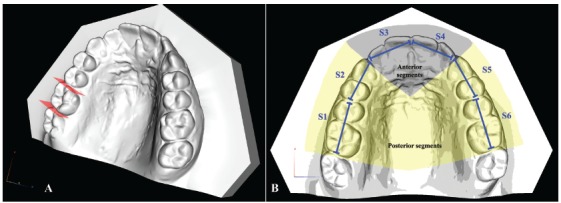
Schematic explanation of tooth size-arch length discrepancy measurements: A) Measurement of mesiodistal width of each tooth; B) The arch is divided into six segments (S1 to S6), which are further pooled into an anterior segment (S3 + S4), a right posterior segment (S1 + S2) and a left posterior segment (S5 + S6).

The digital models were imported into the O3DM^®^ software (Ortolab, Częstochowa, Poland), which was used for the measurements. The tooth size-arch length discrepancy (i.e. crowding) was assessed according to the method described by Lundström.^36^ First, the mesio-distal width of each tooth, excluding the second molars, was measured. Then, the arch was divided into six segments starting from the right first molar to the left first molar, each comprising two teeth at the time (S1= right first molar and second premolar; S2= right first premolar and canine; S3= right lateral and central incisors; S4= left lateral and central incisors; S5= left first premolar and canine; S6= left first molar and second premolar) as shown in Figure 1. Then, the length of each segment was measured, and the sum of the relative teeth widths was calculated. Finally, the difference between the two lengths was calculated. This value was negative in cases with crowding and positive in cases with spacing.

Moreover, a value for tooth size-arch length discrepancy of the anterior region (from the distal contact point of the lateral incisor to the contralateral one) and two values for the left and right posterior regions (from the distal contact area of the first molar to the mesial contact point of the canine) were calculated as well.

### Panoramic radiographs

**Figure 2:**
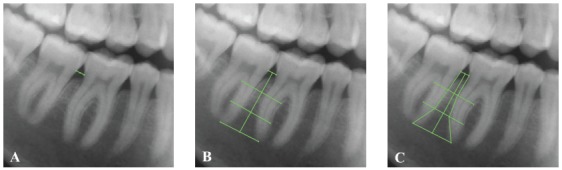
Sequence illustrating the procedure for interradicular space measurements: A) Two points are selected for the CEJ of the adjacent teeth; B) Two points are selected for the roots of the adjacent roots, and the software automatically draws a line perpendicular to the one connecting the CEJs and two perpendicular lines at 1/3 and 2/3 of the root length; C) Four points are added on the 1/3 and 2/3 reference lines to measure the interradicular space.

To measure the interradicular spaces, a specifically designed analysis from PorDios software (PorDios for Windows, Randers, Denmark) was used. Each image was calibrated using the correct DPI. For each interradicular space, one operator manually placed two points at the cemento-enamel junction (CEJ) of the two adjacent teeth and two points at the apex of the adjacent roots. Then, a line connecting the two CEJ points and a line connecting the two apex points were automatically drawn together with the two midpoints of these lines; a third line (midpoint line) connecting these midpoints was then drawn. Two lines were automatically generated perpendicular to the midpoint line, dividing the latter into three equal parts. The interradicular distance was measured along these two lines (i.e. at 1/3 and 2/3 of the root length) (Fig 2). This procedure was repeated for each interradicular space, starting from the mesial aspect of the second molars.

To account for the intrinsic horizontal magnification of the panoramic radiographs, the mesiodistal width of all first molars, first premolars and central incisors was measured both on the panoramic radiographs and on the digital models, to calculate three “magnification factors”: the magnification factor calculated for each first molar was used to adjust the measurement of the interradicular spaces mesial and distal to that first molar; the factor calculated for the first premolar was used for the interradicular spaces mesial and distal to that first premolar; the factor of the central incisor was used for all the interradicular spaces mesial to the canine.

The vertical magnification, also present on the panoramic radiographs, was judged to be negligible since a ratio and not a linear measurement was used to divide the vertical height (1/3 and 2/3) used for the horizontal measurements.

### CBCT

The volumetric data obtained from the NewTom 5G CBCT scanner (QR, Verona, Italy; scan protocol: voxel size 0.3 mm, scanning time 18s, emission time 2.4s, 110kV) were opened with the NNT software (NNT v. 4.6, QR, Verona, Italy) and used to generate custom images. First, the data-set was oriented parallel to the occlusal plane in both the sagittal and the transversal directions; then, using the “broken line” function with a thickness of 9 mm, a panoramic-like image was generated putting a point at the center of each tooth, likely at half the length of the root. Two panoramic-like images (one for the maxilla and one for the mandible) were created, with straight segments between each tooth, to avoid distortions and to ensure the repeatability of the measurements.

The panoramic-like images that were created in this way were imported into the PorDios software, and the same protocol as described before was applied to measure the interradicular spaces.

The data averaged from the panoramic radiographs and CBCTs were used to define two visual maps of the interradicular spaces. In accordance with the literature,^9^ ^,^ ^20^ an interradicular space equal to or exceeding 3.0 mm was considered a “safe zone” for miniscrew placement, whilst an interradicular space of less than 1.6 mm was judged to be unsuitable since it is equal to the diameter of an average miniscrew.^18^ Interradicular spaces measuring less than 3.0 mm but more than 1.6 mm were considered borderline zones, where careful evaluation is needed.

### Error of the method

To evaluate the error of the method, 30 panoramic radiographs, 30 CBCTs and 30 digital models were randomly selected from the whole sample using an online tool (www.randomizer.org), and the measurements were repeated by the same operator after at least one week. For all measurements, Dahlberg’s formula (s = √ (∑d^2^)/2n, where d= difference between the first and second measurements) was used to calculate the standard error on the repeated sets of measurements. Bland-Altman plots were used to check for the intra-observer reliability between the two sets of measurements.^37^

### Statistical analysis

A Shapiro-Wilk normality test was performed for each variable to assess whether the data were normally distributed. Depending on whether the data were normally distributed or not, independent sample T-tests or Mann-Whitney tests were applied to evaluate whether a statistically significant difference between the same variable from the left and right sides was present. If no statistically significant difference was found, the data from the left and the right sides were pooled.

The amount of crowding between the Panoramic group and the CBCT group was compared using the same tests as described above.

To assess whether a correlation between the width of interradicular spaces and tooth size-arch length discrepancy exists, a Pearson correlation (if both variables tested were normally distributed) or a Kendall’s tau test (if one or both variables tested were not-normally distributed) was then performed. The significance level for all tests was set at 0.05.

## RESULTS

The mean age was 16 ± 5.2 years (min = 10, max = 34) for the Panoramic group, and 19 ± 6.1 years (min = 11, max = 34) for the CBCT group. The former included 29 males and 51 females, the latter 30 males and 50 females.

### Error of the method

The average error of the method for measuring tooth size-arch length discrepancy of the individual segments was between 0.18 mm and 0.27 mm in the maxilla and between 0.19 mm and 0.25 mm in the mandible; whilst for the measurement of whole arch tooth size-arch length discrepancy, the error was 0.52 mm for the maxilla and 0.5 mm for the mandible. The average error of the method in measuring interradicular spaces on panoramic radiographs was 0.28 mm (range 0.20-0.34 mm) in the maxilla and 0.48 mm (range 0.32-0.56 mm) in the mandible. The average error of the method in measuring interradicular spaces on CBCTs was 0.30 mm (range 0.21-0.49 mm) in the maxilla and 0.24 mm (range 0.15-0.34 mm) in the mandible. The Bland-Altman plots showed no systematic errors.

### Digital models

In the maxilla, all data were normally distributed, except for tooth size-arch length discrepancy of the posterior right and left segments for patients from both CBCT and Panoramic groups. In the mandible, all data were not normally distributed, except for tooth size-arch length discrepancy of the left posterior segment for the CBCT group.

The measurements of the left and right posterior segments were pooled since no statistically significant differences were found between the two sides both in the mandible and in the maxilla.

Regarding maxillary tooth size-arch length discrepancy, no statistically significant differences were found between the Panoramic and the CBCT groups for the entire arch and all the individual segments; the same applied for the mandibular entire arch and posterior segments (Table 1). On the other hand, a statistically significant difference (*p*= 0.02) in anterior mandibular tooth size-arch length discrepancy was found between the CBCT group and the Panoramic group (Table 1), with the mean difference of only 0.2 mm.

**Table 1:** Descriptive statistics for tooth size-arch length discrepancy (millimeters).

		Panoramic group			CBCT group			
		Mean ± SD	Max	Min	Mean ± SD	Max	Min	p value
Maxilla	Posterior crowding	-0.8 ± 1.5	2.4	-6,7	-0.8 ± 1.5	2,2	-7,3	0.521*
	Anterior crowding	-1.1 ± 1.1	2.6	-4,1	-0.8 ± 1.1	2,4	-4,0	0.062**
	Total crowding of the arch	-3.7 ± 3.6	4.9	-10,8	-3.4 ± 3.6	6,1	-15,5	0.552**
Mandible	Posterior crowding	-1.0 ± 1.3	1,7	-6,4	-1.0 ± 1.3	2,4	-7,3	0.794*
	Anterior crowding	-0.6 ± 0.9	2,7	-2,9	-0.4 ± 1.0	3,9	-4,7	0.022*
	Total crowding of the arch	-3.1 ± 3.4	5,8	-11,0	-2.7 ± 3.2	7,3	-9,4	0.397**

*Mann-Withney U-test; **Independent sample t-test.

### Panoramic radiographs

The magnification factors of panoramic radiographs are reported in Table 2, while the descriptive statistics for interradicular spaces on panoramic radiographs are reported in Table 3.

**Table 2:** Magnification on panoramic radiographs assessed by comparison with digital models (mean ± SD expressed in %).

	Right first molar	Right first premolar	Right central incisor	Left first premolar	Left first molar
Maxilla	27 ± 15	22 ± 16	9 ± 12	25 ± 16	32 ± 16
Mandible	33 ± 17	19 ± 14	19 ± 14	24 ± 15	36 ± 15

**Table 3:** Comparison between measurements on panoramic radiographs with or without calibration through dental casts (mean ± SD in millimeters).

		7_6		6_5		5_4		4_3	
		Coronal 3rd	Apical 3rd	Coronal 3rd	Apical 3rd	Coronal 3rd	Apical 3rd	Coronal 3rd	Apical 3rd
Maxilla	Not calibrated	1.5 ± 0.9	1.0 ± 1.2	2.5 ± 1.0	3.6 ± 1.4*	1.1 ± 0.9	1.7 ± 1.2	0.5 ± 0.7	1.6 ± 1.2
	Calibrated	1.1 ± 0.7	0.8 ± 0.9	1.9 ± 0.8	2.8 ± 1.1	0.9 ± 0.7	1.3 ± 0.9	0.4 ± 0.7	1.3 ± 1.1
Mandible	Not calibrated	3.8 ± 4.1	4.8 ± 5.5	3.5 ± 3.9	5.2 ± 5.7	3.2 ± 3.6	5.6 ± 6.2	1.6 ± 1.9	2.8 ± 3.3
	Calibrated	3.0 ± 0.9*	3.9 ± 1.5*	2.7 ± 0.9	4.1 ± 1.1*	2.8 ± 1.0	4.9 ± 1.4*	1.4 ± 0.9	2.5 ± 1.1

* 3 mm or more of interradicular space; 7_6 = interradicular space between second and first molars; 6_5 = interradicular space between first molar and second premolar; 5_4 = interradicular space between second and first premolars; 4_3 = interradicular space between first premolar and canine; 3_2 = interradicular space between canine and lateral incisor; 2_1 = interradicular space between lateral and central incisors; 1_1 = interradicular space between the central incisors.

In the mandible, all data regarding interradicular spaces were not normally distributed, except for the interradicular spaces between first and second molars at the coronal level. In the maxilla, all data were not normally distributed except for the interradicular spaces between first molar and second premolar at both the coronal and apical third, and between canine and lateral incisor at the apical third. 

The comparisons between the measurements of interradicular spaces on the left and right sides were not statistically significant either in the maxilla or the mandible, except for the interradicular space between the maxillary first and second premolars at the apical third level. However, since this difference was only 0.32 mm, and thus smaller than the measured error of the method (0.34 mm), it was decided to pool the data from the left and right sides for further analysis.

In the maxilla, the only two places where the interradicular space exceeded 3 mm were the interradicular spaces at the apical third level between the canine and lateral incisor, as well as between the two central incisors (Table 3). In the mandible, more than 3 mm were found at the coronal and apical thirds between the second and first molars, between the first molar and second premolar, and between the second and first premolars at the apical thirds (Table 3). 

In the maxilla, a statistically significant positive correlation was found between the tooth size-arch length discrepancy and the presence of interradicular space between the canine and lateral incisor at the coronal and apical thirds, and between the lateral and central incisors at the coronal third (Table 4). In the mandible, the only statistically positive correlation between tooth size-arch length discrepancy and availability of space was found for the space between the central incisors at the coronal third level (Table 4).

**Table 4:** Correlation between tooth size-arch length discrepancy and interradicular spaces on panoramic radiographs

		7_6	6_5	5_4	4_3	3_2	2_1	1_1
Maxilla	Coronal third	0.000	-0.001	0.065	0.107	0.281**	0.180*	0.028
	Apical third	0.049	0.024	0.105	0.038	0.249**	-0.055	0.065
Mandible	Coronal third	-0.011	0.025	0.063	0.039	-0.102	0.147	0.169*
	Apical third	-0.027	0.001	0.019	0.044	-0.058	0.066	0.049

*p<0.05; ** p<0.01; 7_6 = interradicular space between second and first molars; 6_5 = interradicular space between first molar and second premolar; 5_4 = interradicular space between second and first premolars; 4_3 = interradicular space between first premolar and canine; 3_2 = interradicular space between canine and lateral incisor; 2_1 = interradicular space between lateral and central incisors; 1_1 = interradicular space between the central incisors.

### CBCTs

Descriptive statistics for interradicular spaces measured on CBCT images are reported in Table 5. In the maxilla, all data were normally distributed, except for the interradicular spaces between the second and first molars at both the coronal and apical third, between first molar and second premolar at the coronal third, between second and first premolars at the coronal third, between canine and lateral incisor at the coronal third, between the lateral and central incisors at both the coronal and apical third, and between the two central incisors at both the coronal and apical third. In the mandible, all data regarding interradicular space measurements were normally distributed, except for the measurements between the central and lateral incisors at both the coronal and apical third, between the central incisors at both the coronal and apical third levels, and between the first and second molars at the apical third.

**Table 5:** Descriptive statistics for interradicular spaces on CBCTs (mean ± SD in millimeters).

	7_6		6_5		5_4		4_3		3_2	
	Coronal 3rd	Apical 3rd	Coronal 3rd	Apical 3rd	Coronal 3rd	Apical 3rd	Coronal 3rd	Apical 3rd	Coronal 3rd	Apical 3rd
Maxilla	1.1 ± 0.6	1.0 ± 0.7	2.0 ± 0.6	2.1 ± 1	2.0 ± 0.7	2.1 ± 1	1.7 ± 0.7	2.1 ± 0.9	1.8 ± 0.6	2.8 ± 1.2
Mandible	2.7 ± 0.8	3.3 ± 1.3*	2.9 ± 0.7	3.7 ± 1.1*	3.0 ± 0.9*	4.1 ± 1.3*	1.9 ± 0.7	2.6 ± 1.0	1.5 ± 0.5	2.3 ± 1.0

* 3.0 mm or more of interradicular space; 7_6 = interradicular space between second and first molars; 6_5 = interradicular space between first molar and second premolar; 5_4 = interradicular space between second and first premolars; 4_3 = interradicular space between first premolar and canine; 3_2 = interradicular space between canine and lateral incisor; 2_1 = interradicular space between lateral and central incisors; 1_1 = interradicular space between the central incisors.

No differences between the interradicular spaces in the left and right sides in the maxilla as well as the mandible were statistically significant, except for the spaces between the maxillary second and first molars; however, since this difference was only 0.3 mm, a value lying in the same range of both the error of the method and of the voxel dimension, it was considered negligible, and all data from the left and right sides were therefore pooled.

In the maxilla, an interradicular space exceeding 3 mm was present only between the two central incisors at the apical third. In the mandible, an interradicular space exceeding 3 mm was found at the apical third between the first and second molars as well as between the first molar and second premolar, and at both the apical and coronal levels between the first and second premolars (Table 5). 

In the maxilla, a statistically significant positive correlation between tooth size-arch length discrepancy and interradicular space was detected both at the apical and coronal thirds between the first and second premolars, as well as between the canine and lateral incisor, and at the coronal third between the two central incisors. Furthermore, a statistically significant negative correlation was detected for anterior tooth size-arch length discrepancy and interradicular space between the central and lateral incisors at the apical third (Table 6).

**Table 6:** Correlation between tooth size-arch length discrepancy and interradicular spaces on CBCTs.

		7_6	6_5	5_4	4_3	3_2	2_1	1_1
Maxilla	Coronal 3rd	0.018	0.088	0.162**	0.013	0.359**	-0.002	0.162*
	Apical 3rd	0.096	0.097	0.192**	-0.054	0.246**	-0.184*	0.102
Mandible	Coronal 3rd	0.074	0.258**	0.290**	0.175*	-0.016	0.029	0.170**
	Apical 3rd	0.026	0.192*	0.318**	0.075	-0.011	-0.107*	0.075

*p<0.05; ** p<0.01; 7_6 = interradicular space between second and first molars; 6_5 = interradicular space between first molar and second premolar; 5_4 = interradicular space between second and first premolars; 4_3 = interradicular space between first premolar and canine; 3_2 = interradicular space between canine and lateral incisor; 2_1 = interradicular space between lateral and central incisors; 1_1 = interradicular space between the central incisors.

In the mandible, a statistically significant positive correlation between tooth size-arch length discrepancy and interradicular space was detected at the coronal and apical thirds between the first molar and second premolar, as well as between the second and first premolars; at the coronal third between the first premolar and canine, and between the two central incisors at the coronal third. In addition, a statistically significant negative correlation was detected for anterior tooth size-arch length discrepancy and interradicular space between the central and lateral incisors at the apical third (Table 6).

**Figure 3:**
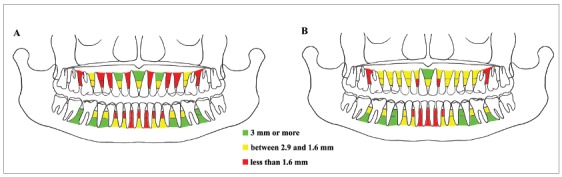
Mapping of interradicular spaces.

**Figure 4:**
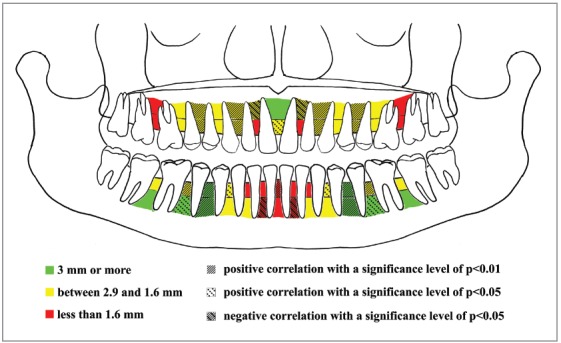
Map showing interradicular spaces measured on CBCTs influenced by the presence of crowding.

All interradicular space measurements from panoramic radiographs and CBCTs and correlations are presented graphically in figures (Figs 3 and 4).

Each interradicular space is divided by a line into two parts (corresponding to the coronal third and apical third measurements), and a color is assigned for each depending on the number of millimeters of space found. Green = 3.0 mm or more; yellow = equal to or more than 1.6 mm; red = less than 1.6 mm. (A) Mapping of interradicular spaces on panoramic radiograph; (B) Mapping of interradicular spaces on CBCT.

## DISCUSSION

The main aim of the present study was to assess the amount of mesiodistal interradicular space between adjacent roots for miniscrew insertion and to correlate these findings with the presence or absence of crowding, in order to develop a simple clinical diagnostic tool for preliminary planning of miniscrew placement. Overall, the error of the method for all measurements of interradicular spaces on both panoramic radiographs and CBCT was small and clinically non-relevant, with the Bland-Altman plots confirming good reliability of the measurements. The measurements of tooth size-arch length discrepancy also showed a small measurement error, confirming that digital models are a reliable method for assessing the presence of crowding and/or spacing in the arch.^38^

The horizontal magnification of the panoramic radiographs was overcome using a calibration method that involved digital models. Magnification assessed on panoramic radiographs was greatest in the lower molar region, whilst the smallest values were found in the upper incisors region (Table 2). These values are smaller than those reported in a previous study (from a minimum of 19% for the maxillary central incisors to a maximum of 55% for the mandibular second molars),^22^ but higher than the magnification values reported by Schnelle et al^9^ (from 2-6% in the anterior region to 22% in the posterior mandible).When measuring interradicular spaces on non-calibrated panoramic radiographs, a general overestimation of the available bone was found. This was in excess of 1 mm in the mandibular molar region compared with the calibrated panoramic radiograph, which underlines the importance of calibrating digital panoramic radiographs, for example by using dental casts, when precise measurement is needed.

The studied sample (Table 1) presented crowding ranging from mild (less than 4 mm) to moderate (from 5 to 9 mm), with only a few cases showing severe (more than 10 mm) crowding.^39^ Overall, baseline characteristics regarding tooth size-arch length discrepancy of both Panoramic and CBCT groups were similar. The differences observed in the present study regarding interradicular space can therefore, indeed, be attributed to the different radiographic techniques. The lower anterior segment is an exception, as a statistically significant difference in the amount of crowding could be observed. However, the difference in crowding between both groups was merely 0.2mm, which is clinically irrelevant. Obviously, a robust way of confirming this statement would be to collect simultaneous panoramic radiographs and CBCTs from the same patients and to compare the images. However, due to ethical reasons that relate to the need of limiting exposure to ionizing radiation, it is not possible to prospectively recruit a sample where both radiographic techniques are employed at the same time on the same patients. For the same reason, it is almost impossible to retrospectively retrieve such a group of patients where both panoramic radiograph and CBCT were taken at the same point in time.

The maps (Fig 3A and 3B) can be used to estimate the optimal sites for miniscrew insertion. In general, slightly more interradicular space was found in the mandible than in the maxilla. 

In a previous study about “safe zones” for miniscrew placement based on panoramic radiographs,^9^ the areas between premolars both in the mandible and maxilla were discarded due to a high distortion risk. In the same study, it was found that sites with more than 3 mm of interradicular space in the maxilla were evident between the first molar and second premolar, the canine and lateral incisor, and the central incisors; whereas in the mandible, these sites were between the second and first molar, the first molar and second premolar, and the canine and lateral incisor. These results are in accordance with those of the present study, except for the space between the maxillary first molar and the second premolar, as well as between the mandibular canine and lateral incisors, where smaller spaces were detected.

Poggio et al^20^ evaluated interradicular spaces at different levels of the alveolar crest on CBCTs. The greatest amount of space in the maxilla was found between the second and first premolars, between the first premolar and canine, and to a lesser extent between the first molar and second premolar; in the mandible, with the exception of the space between the first premolar and canine, there was generally a good amount of space. The results for mandibular spaces are consistent with those of the present study, whilst in the maxilla the interradicular spaces found in the present study were smaller than those previously reported. However, it should be considered that the reference points in the present study were sampled at different levels, which increases precision and may help explain the different results.

Despite the heterogeneity of the considered publications, a systematic review^11^ reported general agreement regarding the best sites for miniscrew placement: the areas between the first and second molars, the first molar and second premolar both in the maxilla and in the mandible were indicated as the best locations. In the present study, however, the posterior region of the maxilla, and the space between the second and first molars in particular, showed a small amount of available bone, thus contradicting what was found in the literature. Overall, panoramic radiographs underestimated the available interradicular spaces compared with CBCT, which is considered the gold standard for linear measurements.^40^ Two exceptions were the space at the coronal third between the first and second molars in the mandible, and the space at the apical third between the maxillary canine and lateral incisor. The latter can probably be explained because at that point the arch displays an increased curvature and panoramic radiographs images therefore present greater distortion.^41^

To test the hypothesis that assessment of tooth size-arch length discrepancy can be used as a preliminary clinical tool for the evaluation of miniscrew insertion, the correlation between the amount of dental crowding and the presence of sufficient interradicular space for miniscrew insertion was assessed as well in the present study. In general, tooth size-arch length discrepancy measured at the crown level seems to be related to the amount of interradicular space. Schnelle et al^9^ repeated interradicular space measurements on post-orthodontic treatment panoramic radiographs of patients assessed before treatment: in this way, they assessed whether having roots that are parallel and aligned following orthodontic treatment generally ensures a greater number of available interradicular spaces. In particular, they observed that the availability of ≥ 3 mm of bone increased at the space between the maxillary canine and lateral incisor, and between the mandibular canine and lateral incisor. In the present study, a statistically significant correlation between tooth size-arch length discrepancy and interradicular spaces measured on panoramic radiograph was found for the space between the canine and lateral incisor in the maxilla. The presence of this correlation is particularly important for interradicular areas where a suitable amount of space for miniscrew insertion is usually found; however, for a patient who has crowding, it should be expected that the interradicular space would be less than usual.

In their review, AlSamak et al^11^ proposed the use of “safe zone” maps provided by the literature to define guidelines for miniscrew insertion, arguing that they would thereby avoid radiographs, at least for those sites that have proven to be favorable. Indeed, sometimes clinical examination alone is appropriate for evaluating miniscrew insertion sites. Considering also the results of Landin et al,^35^ this task can be reasonably achieved. In the present study, the value of “safe zone” maps was improved by additional data from tooth size-arch length discrepancy; maps of average interradicular space are important, but the presence of crowding or spacing may substantially change the actual space available.

**Figure 5:**
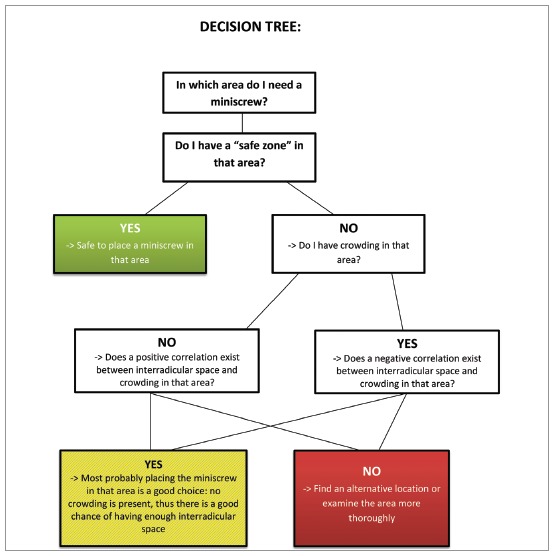
Decision tree to be used together with the map in Figure 4, to help clinicians evaluate the possibility of miniscrew insertion from the outset of orthodontic treatment planning.

**Figure 6:**
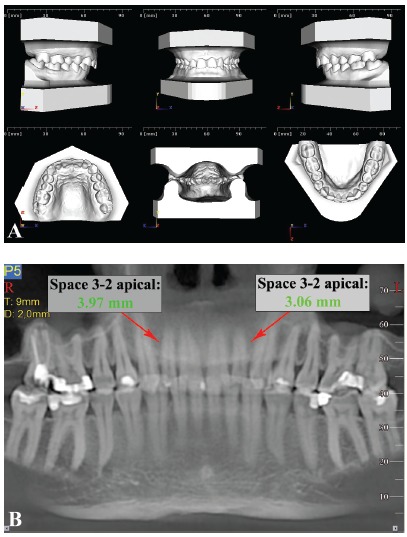
Clinical example of a deep bite case where miniscrews inserted between lateral incisors and canines could be used for intrusion of the maxillary anterior segment. Miniscrews are planned distal to the maxillary lateral incisors; according to the map, the chosen insertion sites are marked as yellow, but with a strong correlation between crowding and interradicular space. Since there is no crowding, the interradicular space is assumed to be sufficient and miniscrews can be considered. Indeed, the CBCT confirms this deduction. A) Digital models showing no crowding in the maxillary arch; B) Panoramic-like image of the maxilla.

**Figure 7:**
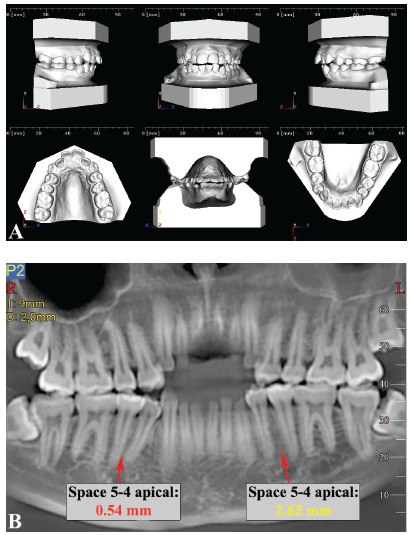
Clinical example of a patient with moderate crowding. In the mandible, for example, miniscrews between the first and second premolars could provide absolute posterior anchorage to solve crowding and incisal protrusion. According to the map, the interradicular spaces between the first and second premolars are marked as green sites, but with a strong correlation between crowding and interradicular space. Therefore, less space for miniscrew insertion may be expected in the case of crowding. In this case, on the right side, which is more crowded than the left, the CBCT confirms that inserting a miniscrew is not possible. A) Digital models showing crowding in both maxillary and mandibular arches; B) Panoramic-like image of the mandible.

The last map provided (Fig 4), derived from CBCT interradicular spaces measurements and correlations with tooth size-arch length discrepancy, can be used during the initial stage of orthodontic treatment planning in combination with the measurement of the tooth size-arch length discrepancy. This map can be used to evaluate the probability of sufficient space when increased crowding is present: the space can indeed be different from what is commonly found in the literature. Therefore, the map can be used in combination with tooth size-arch length discrepancy assessment in light of the ALARA principle, whereby redundant radiographic investigation of the patient may be avoided. The map may also be used to unveil different biomechanics to bypass those inconvenient spaces, or even to choose from the outset to use 3D radiographic examination that allows more comprehensive evaluation of the desired insertion sites. To help clinicians in this process, a decision tree based on the maps of safe zones and the map of correlations has been proposed (Fig 5). Nevertheless, further studies are needed to validate the suggested method in a clinical environment. To illustrate how the decision tree could help in clinical scenarios, two examples are provided (Figs 6 and 7). 

Fewer correlations between tooth size-arch length discrepancy and interradicular spaces were found on panoramic radiographs with respect to CBCTs. An explanation for this finding could be that smaller interradicular spaces were measured overall on panoramic radiographs, and therefore the smaller range of values could have limited the correlation. 

Although with Pearson correlation or Kendall’s tau tests a value of +1 means a perfect positive correlation and -1, a perfect negative correlation, in this particular case, a positive correlation coefficient means that when crowding increases, interradicular space decreases, since a positive (interradicular space) and a negative value (dental crowding) were correlated.

Surprisingly, when correlating interradicular spaces measured on CBCTs and tooth size-arch length discrepancy, a negative correlation was found at the apical level of the interradicular space between the lateral and central incisors in both the maxilla and the mandible, which means that for these regions, more crowding results in more interradicular space. The reason for this finding may relate to the divergence of the roots where crowding is present; however, further investigations need to be performed to verify this finding.

It is important to underline that single interradicular spaces were correlated with the tooth size-arch length discrepancy of the entire relative segment (anterior or posterior) and not with a value of tooth size-arch length discrepancy between the two adjacent teeth relative to that interradicular space. This procedure was chosen to reflect what is usually applied in clinical practice, where tooth-by-tooth assessment of crowding is meaningless. 

“Safe zone” maps should be used in combination with the map showing which interradicular spaces are correlated with dental crowding (Fig 4). This combination facilitates the preliminary planning of miniscrew insertion before choosing which radiographs to prescribe, thereby making it possible to avoid unnecessary ionizing radiation. 

## CONCLUSIONS

1. The use of digital models to calibrate panoramic images constitutes a valuable tool, while direct horizontal measurements on non-calibrated panoramic radiographs lack precision.
2. Overall, compared with CBCT, panoramic radiographs underestimate the actual interradicular space, hindering the use of miniscrews when in reality insertion would be possible, provided that the amount of crowding is the same.
3. The findings of this study result in a new “safe zone” map. The best areas for miniscrew insertion are between the upper central incisors and the interradicular spaces from the mandibular second molar to the mandibular first premolar.
4. The map correlating interradicular spaces to tooth size - arch length discrepancy may surpass previously published “safe zones” when crowding is present. Used in combination with the decision tree and the measured tooth size - arch length discrepancy, this map may help clinicians in the preliminary planning of miniscrew insertion and in choosing appropriate radiographs, when necessary.
